# High Expression of MicroRNA-196a is Associated with Progression of Hepatocellular Carcinoma in Younger Patients

**DOI:** 10.3390/cancers11101549

**Published:** 2019-10-13

**Authors:** Shen-Yung Wang, Chih-Li Chen, Yu-Chen Hu, Yi Chi, Yen-Hua Huang, Chien-Wei Su, Wen-Juei Jeng, Yuh-Jin Liang, Jaw-Ching Wu

**Affiliations:** 1 Institute of Clinical Medicine, National Yang-Ming University, Taipei 11221, Taiwan; 2 Division of Gastroenterology and Hepatology, Department of Medicine, MacKay Memorial Hospital, Taipei 10449, Taiwan; 3 School of Medicine, College of Medicine, Fu-Jen Catholic University, New Taipei City 24205, Taiwan; 4 Department of Chemical Engineering, National Tsing Hua University, Hsinchu 30013, Taiwan; 5 Frontier Research Center on Fundamental and Applied Sciences of Matters, National Tsing Hua University, Hsinchu 30013, Taiwan; 6 Medical Research Department, Taipei Veterans General Hospital, Taipei 11217, Taiwan; 7 Institute of Biomedical Informatics, National Yang-Ming University, Taipei 11221, Taiwan; 8 Center for Systems and Synthetic Biology, National Yang-Ming University, Taipei 11221, Taiwan; 9 Cancer Progression Research Center, National Yang-Ming University, Taipei 11221, Taiwan; 10 Faculty of Medicine, School of Medicine, National Yang-Ming University, Taipei 11221, Taiwan; 11 Division of Gastroenterology and Hepatology, Department of Medicine, Taipei Veterans General Hospital, Taipei 11217, Taiwan; 12 Department of Gastroenterology and Hepatology, Chang Gung Memorial Hospital, Linkou Branch, Taoyuan City 33305, Taiwan; 13 College of Medicine, Chang Gung University, Taoyuan City 33302, Taiwan

**Keywords:** liver cancer, microRNA, recurrence, stemness

## Abstract

MicroRNAs are small RNAs involved in various biological processes and cancer metastasis. miR-196a was associated with aggressive behaviors in several cancers. The role of miR-196a in hepatocellular carcinoma (HCC) metastasis remains unknown. This study aimed to examine the role of miR-196a in HCC progression. Expression of miR-196a was measured in 83 human HCC samples. The HCC patients with high miR-196a expression had younger ages, lower albumin levels, higher frequency with alpha-fetoprotein (AFP) levels ≥20 ng/mL, more macrovascular invasion, and non-early stages. Kaplan–Meier analysis showed that high miR-196a expression was associated with lower recurrence-free survival. Knockdown of miR-196a decreased transwell invasiveness, sphere formation, transendothelial invasion, and Slug, Twist, Oct4, and Sox2 expression, suppressed angiogenesis, and reduced sizes of xenotransplants and number of pulmonary metastasis. Down-regulation of miR-196a decreased Runx2 and osteopontin (OPN) levels. Knockdown of Runx2 in vitro resulted in comparable phenotypes with miR-196a down-regulation. Restoration of Runx2 in miR-196a-knockdown HCC reverted tumor phenotypes. This study showed that high expression of miR-196a is associated with HCC progression in a subset of younger patients. miR-196a mediates HCC progression via upregulation of Runx2, OPN, epithelial–mesenchymal transition (EMT) regulators, and stemness genes. We proposed that miR-196a can be used as a prognostic marker and a potential therapeutic target.

## 1. Introduction

Hepatocellular carcinoma (HCC) is the most lethal and prevalent type of liver cancer; it is also the sixth most common cancer worldwide, with 841,080 new cases every year. Among cancers, HCC ranks third for mortality, and an estimated 781,631 cases die of HCC in the world annually [1]. Despite enormous efforts to improve clinical treatment, HCC remains a major carcinoma with high mortality. Poor differentiation, large tumor size, portal invasion, and intrahepatic metastasis are known to shorten the disease-free survival in this carcinoma [2]. Surgical resection is one of the methods of a potential cure. However, recurrence remains high and is the major cause of mortality of HCC patients [2]. Elucidation of the molecular mechanisms, which induce highly invasive behavior of HCC, is the most important issue for developing strategies to prevent and treat HCC metastasis.

MicroRNAs (miRNAs) are small RNAs of 18–25 nucleotides in length that are involved in the regulation of a variety of biological processes including developmental timing, signal transduction, apoptosis, cell proliferation, and tumorigenesis [3]. miRNAs function to silence gene expression through imperfect base-pairing with cognate transcripts. Since RNA silencing mediated by miRNA does not require perfect sequence complementarity, one miRNA can target multiple different mRNAs [3]. More than 2000 mature human miRNAs have been discovered to date [4]. Murakami et al. first reported the differences of miRNA expression patterns between HCC tumor and nontumorous tissues and a significantly different pattern of miRNA expression between HCC and liver cirrhosis samples [5]. miRNAs are reported as important mediators in HCC progression and metastasis [6].

Upregulation of miR-196a was shown to be associated with large tumor size, advanced stage, and poor survival in gastric cancer [7]. Luthra et al. reported that miR-196a was abundantly expressed in human esophageal adenocarcinoma samples as well as esophageal cancer cell lines [8]. Downregulation of miR-196a reduced the level of osteopontin (OPN) produced by bone marrow stem/stoma cells [9]. Reports from several groups showed that OPN was involved not only in the bone regeneration, but also in the cancer metastasis [10]. The expression of OPN significantly correlates with clinicopathological features of HCC patients with capsular infiltration, vascular invasion, lymph node metastasis, and tumor-node-metastasis (TNM) stages [11,12]. However, there is no report that discusses the role of miR-196a in HCC tumorigenesis or metastasis. In this study, we investigated the role and clinical significance of miR-196a in HCC, and the interaction between miR-196a and epithelial–mesenchymal transition (EMT) regulators and stemness genes. Furthermore, our results indicated that miR-196a is pivotal in HCC progression by modulating the level of Runx2 and OPN.

## 2. Results

### 2.1. Correlation of MiR-196a Expression with Clinical–Pathological Parameters and HCC Progression

A total of 83 patients with HCC and hepatitis B virus (HBV) infection received tumor resection were recruited in this study. As shown in Table 1, the median age of patients was 51 years old (range, 33 to 74 years old). There were 70 male patients (accounted for 84.3%). Nearly half of the patients had liver cirrhosis (*n* = 38, 45.8%). The tumor stage was determined according to the 7th edition of the American Joint Committee on Cancer (AJCC) TNM staging system [13]. Among these patients, 42 were stage I, 17 were stage II, 22 were stage III, and 2 were stage IV. The median size of resected HCC was 4 cm (interquartile range, 2.5 to 7.3 cm). The median follow-up period after surgical resection was 42.0 months (range, 1 to 75 months).

To determine the relationship between miR-196a and clinical characteristics of HCC, the 83 patients were divided into groups of high and low miR-196a expression, defined by a cutoff using the median level of expression. The age of the group with high miR-196a expression was younger than the lower expression group (median 47.5 years old vs. 56 years old, *p* = 0.0369). The serum level of albumin was significantly lower in the high-expression group (range, 3.4 to 4.1 g/dL vs. 3.8 to 4.3 g/dL, *p* = 0.0386). High expression of miR-196a was more frequently associated with serum level of alpha-fetoprotein (AFP) ≥20 ng/mL (63.6% vs. 32.4%, *p* = 0.01). The group with high miR-196a expression had significantly more macrovascular invasion than those with low expression (19% vs. 2.4%, *p* = 0.0375).

The high expression level of miR-196a was not associated with host factors of gender or liver cirrhosis. HBV virological factors including genotype, viral loads, and HBeAg status were not significantly different between groups of high or low expression of miR-196a. Several tumor factors including tumor size, tumor grading, and multinodularity of HCC were similar in both groups. Although there was more macrovascular invasion in the high expression of miR-196a group, the percentage with microvascular invasion did not significantly differ when comparing the groups.

The factors associated with recurrence of HCC were investigated. Early tumor stage was correlated with significantly less recurrence. The presence of microvascular invasion was higher in HCCs with recurrence compared with those without recurrence (51.2% vs. 23.5%, *p* = 0.0255, Table S1). However, macrovascular invasion did not vary between HCC with or without recurrence. Age, gender, or liver cirrhosis was not associated with HCC recurrence. Some studies have suggested diabetes mellitus may play a role in advanced HCC [14]. However, diabetes mellitus was not associated with HCC recurrence in our cohort. HBV viral factors were not significantly different among groups with or without recurrence, despite the presence of HBV genotype C, indicating a slight trend toward HCC recurrence (*p* = 0.087). The distributions of tumor size, tumor differentiation, multinodularity, and AFP level did not significantly contribute to HCC recurrence (Table S1).

The univariate and multivariate analyses for evaluating factors associated with recurrence are summarized in Table 2. The univariate analysis showed that microvascular invasion and high expression of miR-196a were significant factors for the higher incidence of recurrence (Table 2). The crude hazard ratio of microvascular invasion was 3.429 (95% confidence interval (CI), 1.831 to 6.419) for HCC recurrence, and that of high expression of miR-196a was 2.124 (95% CI, 1.148 to 3.929). The multivariate Cox regression analysis also revealed that higher expression of miR-196a was an independent predictor for HCC recurrence (Table 2). The adjusted hazard ratio of high expression of miR-196a was 2.395 (95% CI, 1.207 to 4.752). The microvascular invasion was also an independent predictor for HCC recurrence. Other viral or tumor factors such as HBV viral load or genotype, multinodular HCC, or macrovascular invasion did not show statistically significant association with the recurrence of HCC.

**Figure 1:**
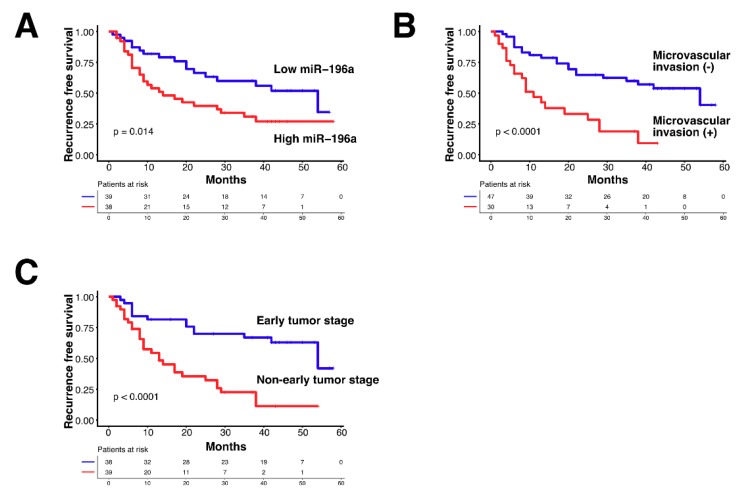
Factors associated with recurrence-free survivals in hepatocellular carcinoma (HCC) patients who underwent tumor resection. (**A**) A Kaplan–Meier method compares cumulative recurrence-free survivals between HCC patients with high or low miR-196a expression. (**B**) Comparison of recurrence-free survivals between HCC patients with or without microvascular invasion. (**C**) Comparison of recurrence-free survivals between HCC patients with early or non-early tumor stages.

The cumulative incidence of recurrence analyzed by the Kaplan–Meier method showed that recurrence-free survival was significantly different between the high and low expression of miR-196a (Figure 1A, log-rank test *p* = 0.014). The presence of microvascular invasion is also a strong factor for the cumulative incidence of recurrence (Figure 1B, log-rank test *p* < 0.0001). TNM stage I had much less recurrence than other stages. Non-early tumor stage (TNM stage II and III and IV) had a shorter time to recurrence compared with that of stage I (Figure 1C, log-rank test *p* < 0.0001).

### 2.2. Downregulation of MiR-196a Expression Impaired Sphere Formation and Invasiveness

To explore the role of miR-196a in the progression of HCC, the RNA expression levels of miR-196a in various HCC cell lines, including Hep3B, PLC, Mahlavu, SNU449, Huh7, and HepG2, were examined by reverse transcription–real-time quantitative PCR. We found that expression of miR-196a was upregulated in Hep3B, PLC, Mahlavu, SNU449, and Huh7, which was about 25- to 80-fold higher than that in HepG2 (Figure S1). The expression of miR-196a was scarce in normal liver tissue (Figure S1). These findings suggest that miR-196a was abundantly expressed in multiple HCC cell lines, including HCC cell lines originated from HBV-related HCC (Hep3B, PLC, and SNU449).

**Figure 2:**
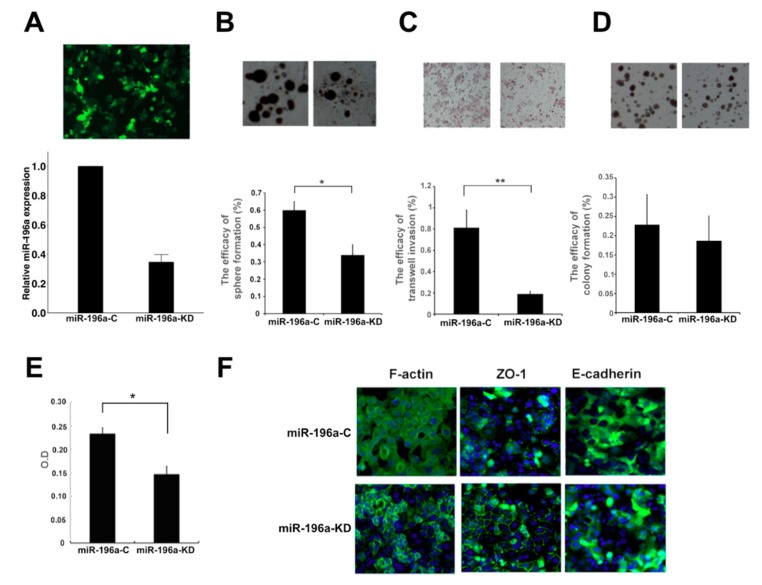
The effects of miR-196a knockdown on self-renewal, transwell-invasion, and colony formation of PLC cells. (**A**) Suppression knockdown (KD) of miR-196a after infection with lentiviral vectors expressing antisense miR-196a (miR-196a-KD) compared with that with negative control vectors (miR-196a-C). (**B**–**D**) Knockdown of miR-196a significantly decreased sphere formation of PLC cells (**B**) and transwell-invasion (**C**), but not colony formation (**D**). (**E**) Suppression of miR-196a decreased transendothelial migration capabilities of PLC. (**F**) miR-196a downregulation altered cytoskeleton organization, ZO-1 localization, but not E-cadherin. *p < 0.05, **p < 0.01. Original magnification, ×100 (**A**), ×50 (**B**), ×100 (**C**), ×25 (**D**), ×400 (**F**).

PLC is an HBV-related HCC cell line that is compatible with our HCC patient cohort, and it has a high expression of miR-196a. Therefore, the PLC cell line was used to study the effects of knocking down miR-196a using the lentivirus vector carrying antisense miR-196a. Compared with the vector infection, the expression of miR-196a was suppressed to 40% by lentivirus-mediated miR-196a knockdown in PLC cells (Figure 2A). To evaluate the effect of miR-196a in tumor progression, invasion assay, sphere formation, and transendothelial invasion assay were performed using PLC cells with or without miR-196a knockdown. As shown in sphere formation, a way to assess self-renewal abilities of cancer stem cells, knockdown of miR-196a decreased the sizes of spheres of PLC cells (Figure 2B), the invasiveness of PLC cells (Figure 2C), but not colony formation (indicating cell survival and proliferation) (Figure 2D). Transendothelial invasion is a major step of cancer metastasis. Compared with the PLC cells infected with control vector, the knockdown of miR-196a resulted in fewer tumors cells migrating across the vascular endothelium (Figure 2E). In support of the alteration of HCC invasion ability, miR-196a knockdown resulted in reduced stress fiber formation and actin staining in the cytoplasm of PLC cells (Figure 2F, the left panel), and higher levels of membranous zonula occludens-1 (ZO-1), a tight junction-associated protein (Figure 2F, the middle panel). However, E-cadherin expression was not significantly altered in PLC cells with or without miR-196a knockdown (Figure 2F, the right panel). Consistent results that miR-196a knockdown resulted in the reduction of sphere formation, transwell invasiveness, and no significant change in colony formation were seen in another HCC cell line SNU449 (Figure S2).

### 2.3. Knockdown of MiR-196a Decreased Angiogenesis and Lung Metastasis of HCC

**Figure 3:**
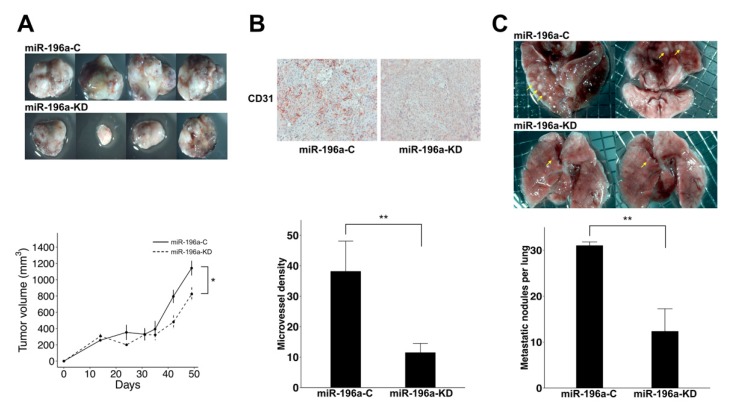
miR-196a downregulation impairs tumor growth of xenotransplants and pulmonary metastasis of PLC cells. (**A**) The upper row shows harvested tumors from mice that received subcutaneous implantation of PLC cells without knockdown, and the lower row shows tumors from xenografts of implanted PLC cells with miR-196a knockdown (top). The periodically measured sizes of HCC xenografts had a trend of lower growth curves for PLC cells with miR-196a knockdown (bottom). (**B**) Knockdown of miR-196a led to the suppression of the intratumoral microvessels, assessed by CD31-positive tubes (bottom, 200× magnification, **p < 0.01). (**C**) The upper row shows lungs from mice that received PLC without knockdown, and the lower row shows lungs from mice receiving PLC with miR-196a knockdown, which showed a decreased extent of pulmonary metastasis (yellow arrows). The diameter of the mesh opening was 0.2 mm (top). The number of metastatic nodules in the sectioned lungs was significantly decreased after knockdown of miR-196a (**p < 0.01).

To examine the impacts of miR-196a on tumorigenesis activity of HCC cell lines, PLC cells with or without miR-196a knockdown were implanted subcutaneously over bilateral flanks of non-obese diabetic–severe combined immunodeficiency (NOD/SCID) mice as a xenograft model. The periodically measured sizes of the xenografts showed that downregulation of miR-196a suppressed the tumor growth of PLC cells (Figure 3A). The harvested tumors derived from miR-196a-downregulated PLC cells were smaller than those from the PLC controls (Mean 823.9 mm^3^ vs. 1142.6 mm^3^, *p* = 0.05, Figure 3A). The results of immunohistochemistry (IHC) using anti-CD31 revealed that downregulation of miR-196a significantly reduced the number of CD31-positive three-dimensional tubes within the subcutaneous xenografts of PLC (Figure 3B). The measured intratumoral microvessel density was significantly decreased in the xenografts derived from miR-196a-KD PLC as compared with those derived from miR-196a-C (Mean 9.9/field vs. 38.1/field, *p* = 0.01, Figure 3B). Because miR-196a knockdown impaired the transendothelial invasion of PLC (Figure 2E), we further examined the metastasis ability in mice using an injection of tumor cells into mouse tail veins. The numbers of metastatic pulmonary lesions were significantly decreased after knockdown of miR-196a (Mean: 12.3/lungs vs. 31.0/lungs, *p* = 0.01, Figure 3C). The results indicated that decreased expression of miR-196a led to the suppression of pulmonary metastasis.

### 2.4. Knockdown of MiR-196a Modulated EMT Regulators and Stemness Regulators of HCC

**Figure 4:**
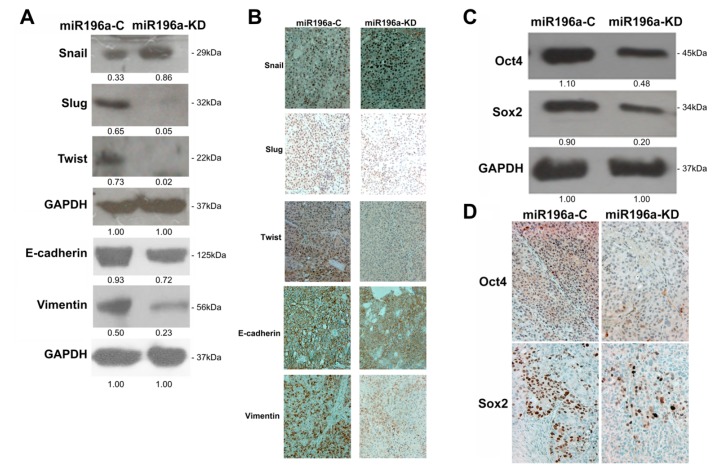
Knockdown of miR-196a modulated epithelial–mesenchymal transition regulators and stemness genes in PLC cells. (**A**) Western blotting indicated that miR-196a downregulation led to a decrease of Slug and Twist. (**B**) Immunohistochemistry revealed that the levels of Slug and Twist were reduced in the xenografts derived from PLC-miR196a-KD compared with those from PLC-miR196a-C (original magnification, ×200). (**C**) Western blotting showed that downregulation of miR-196a reduced the levels of Oct4 and Sox2 in PLC. (**D**) Immunohistochemistry showed that downregulation of miR-196a reduced Oct4 and Sox2 expression in the subcutaneous xenografts derived from miR196a-KD PLC cells compared with those from miR196a-C PLC cells (original magnification, ×200).

EMT is an important process for tumor progression and cancer metastasis in the HCC progression [15]. As shown in Figure 2B,E, and Figure 3, our in vitro and in vivo studies indicated that miR-196a might be involved in regulating the metastasis of PLC. Thus, we investigated whether miR-196a affected EMT regulators. The results of Western blotting indicated that knockdown of miR-196a downregulated the expression of Slug, Twist, and Vimentin in the PLC HCC cell line (Figure 4). On the other hand, we also found that Snail was upregulated in the miR-196a-knockdown PLC cells (Figure 4A). The findings were also confirmed with in vivo xenografts derived from HCC cell line by IHC analysis, with the corresponding decrease of Slug, Twist, and Vimentin staining in the miR-196a-suppressed tumors (Figure 4B, Figure S3A). The properties of stemness are important for tumor self-renewal and cancer metastasis in the HCC progression. Oct4 and Sox2 were reported as transcription factors involved in maintaining the stemness properties [16]. The knockdown of miR-196a reduced the levels of Oct4 and Sox2 both in PLC (Figure 4C) and in the corresponding staining in subcutaneous xenografts derived from miR-196a-knockdown PLC cells (Figure 4D, Figure S3B).

### 2.5. MiR-196a Promotes HCC Invasiveness by Regulating Runx2 and OPN

**Figure 5:**
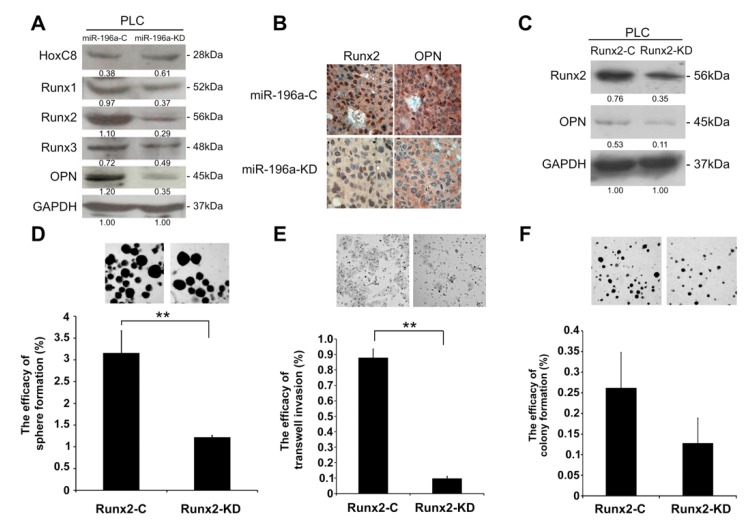
Runx2 knockdown reduced self-renewal and transwell invasion of PLC cells. (**A**) Western blot revealed that miR-196a downregulation decreased Runx family and osteopontin (OPN) expression of PLC cells. (**B**) Immunohistochemistry showed that the expression of Runx2 and OPN were decreased in xenografts derived from miR-196a-KD (original magnification, ×400). (**C**) Western blot revealed the levels of Runx2 and OPN in Runx2 knockdown (Runx2-KD) and control (Runx2-C) PLC cells. (**D**–**F**) Knockdown of Runx2 significantly decreased the sphere formation of PLC cells (**D**) and transwell invasion (**E**), but not colony formation (**F**) (original magnification, ×50 (**D**), ×100 (**E**), ×25 (**F**), **p < 0.01).

Previous studies have shown that miR-196a upregulates the expression of OPN through downregulation of HoxC8 [9]. However, our study revealed that the level of HoxC8 in the PLC with knockdown of miR-196a is comparable to those in the control cells (Figure 5A, Figure S6A). Runx2, a vital factor for driving immature osteoblasts to maturation, also up-regulates OPN. We assumed that miR-196a was involved in the progression of HCC by modulating the level of OPN by increasing the expression of Runx2. Thus, we performed Western blotting to explore whether miR-196a downregulation affects the expression of Runx family and OPN. The results indicated that the levels of Runx2 and OPN of miR-196a-KD were reduced as compared with those of miR-196a-C (Figure 5A, Figure S2B, Figure S6A). IHC also revealed the downregulation of Runx2 and OPN in the xenografts derived from miR-196a-KD as compared with those of xenografts derived from miR-196a-C (Figure 5B, Figure S3C). Western blotting also indicated Runx2 knockdown resulted in a decrease of OPN (Figure 5C, Figure S6B). Knockdown of Runx2 in the PLC also led to impaired sphere formation (Figure 5D), invasiveness (Figure 5E), but not colony formation (Figure 5F) of PLC. Also, Runx2 downregulation reduced stress fiber formation and actin staining in PLC (Figure S4, the left panel), increased higher levels of ZO-1 (Figure S4, the middle panel), but E-cadherin expression remained comparable (Figure S4, the right panel).

**Figure 6:**
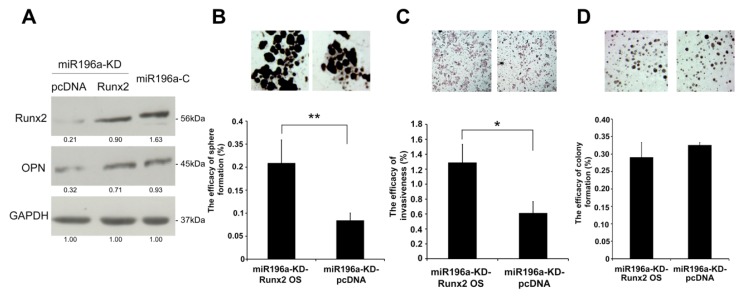
Restoration of Runx2 in miR-196a-downregulated PLC cells increased self-renewal and invasiveness. (**A**) Western blot confirmed the rescue of expression of Runx2 and osteopontin (OPN) after expression of Runx2 in miR-196a-downregulated PLC cells. (**B**–**D**) Restoration of Runx2 in miR-196a-downregulated PLC cells rescued the efficacy of sphere formation (**B**) and invasiveness (**C**), but not colony formation (**D**) (original magnification, ×50 (**B**), ×100 (**C**), ×25 (**D**), *p < 0.05, **p < 0.01).

Overexpression of Runx2 in miR-196a-downregulated PLC was performed to investigate if Runx2 can rescue the phenotypes. Western blotting was performed to confirm the rescue of the level of Runx2 (Figure 6A, Figure S6C). Transient expression of Runx2 in miR-196a-downregulated PLC can rescue the efficacy of sphere formation (Figure 6B) and invasiveness (Figure 6C), but not colony formation (Figure 6D). Our observation suggests that miR-196a might control the progression of HCC by regulating the levels of Runx2 and OPN.

## 3. Discussion

Budhu et al. [6] reported 20 miRNAs related to HCC metastasis. However, miR-196a was not among these, which might be due to the use of different HCC groups with different ages. To our knowledge, the results of this study are the first to show that miR-196a is an independent predictor for HCC progression and postoperative recurrence in a subset of patients with younger age. Of note, HCC patients with high miR-196a expression were significantly younger (8.5 years younger in the median age of the high vs. the low expression groups). Interestingly, HCC patients with high miR-196a expression tended to have significantly higher AFP expression, which is a marker of HCC stem cells [17]. Moreover, high expression of miR-196a is associated with upregulated Oct4, Sox2, and increased sphere formation in vitro and larger sizes of HCC xenotransplants in vivo, indicating increased expression of stemness genes and self-renewal. In our previous report, younger HCC patients have more embryonic stem cell gene expression, macroscopic venous invasion, and poor prognosis [18]. In the current study, HCC patients with high miR-196a expression also had significantly higher AFP levels and more macroscopic venous invasion, consistent with previous reports with much larger numbers of young HCC patients [19,20,21]. In vitro and in vivo studies in the current study indicate that HCC cells with higher miR-196a expression had higher trans-endothelial invasion capability, as well as higher invasion ability both in vitro and in vivo, which may explain the greater macroscopic invasion and high recurrence rates in these patients. Taken together, miR-196a likely plays an important role in HCC progression in a subset of HCC patients with relatively younger age, higher AFP levels and stemness gene expression, more macroscopic venous invasion, and poor prognosis.

Upregulation of miR-196a was shown to be associated with prognosis of various cancers [7,8]. However, the mechanisms for this remain unclear. In addition to the discovery of the correlation of miR-196a with HCC recurrence, this study provides molecular mechanisms for this correlation. In the current study, the knockdown of miR-196a changed tumor phenotypical behaviors, including decreased invasiveness, sphere formation, and transendothelial invasion capabilities and decreased angiogenesis and decreased pulmonary metastasis in vivo. Further investigations showed reduced EMT expression in Slug, Twist, vimentin, and stemness genes of Oct4 and Sox2 in miR-196a-knockdown cells. To our knowledge, this is the first report that miR-196a plays an important role in the progression of HCC via regulation of EMT and stemness genes. Cell migration is a crucial process in cancer progression [22]. In our previous studies, EMT correlates well with early recurrence related to metastasis within 2 years after operation [15]. Recently, we found that LEF1 reciprocally transactivates EMT regulators and stemness genes and plays a key role in postoperative HCC recurrence [23].

A group of transcription factors forms the backbone of the EMT cascade, and several lines of evidence show that miRNAs are heavily involved in mesenchymal transformation, by suppressing the expression of different groups of transcription factors, or otherwise acting as their functional mediators in orchestrating EMT [24,25]. Twist and Slug are transcription factors involved in the EMT cascade. Expression of the EMT transcriptional factor, Twist, was reported to be associated with aggressive behaviors of cancers and participated in miRNA-mediated EMT regulation [15,25]. The Slug was shown to be pivotal in cancer-associated EMT and enhancing EMT in conjunction with miRNAs in a self-reinforcing double feedback loop [15,26]. This study indicates that knockdown of miR-196a results in suppression of the major regulators of EMT, Twist and Slug, which implies that miR-196a is an important mediator of EMT. Thus, our data suggest that miR-196a orchestrates the EMT process by regulating the levels of Twist and Slug. Although Twist and Slug expression was decreased after miR-196a knockdown, Snail was not suppressed. The cause of differential regulation of EMT regulators by miR-196a remains to be further investigated. Nevertheless, xenotransplants and pulmonary metastasis using miR-196a-knockdown PLC cells were still significantly reduced in sizes and numbers compared with those using wild-type cells. Interestingly, the current study finds differential regulation of ZO-1 and E-cadherin, showing that knockdown of miR-196a did not significantly increase E-cadherin expression representing adherans junctions, but significantly increased the expression of ZO-1 representing tight junctions [27]. Overexpression of ZO-1 was suggested to suppress proliferation and migration of liver cancer cells and induce cell cycle arrest [28]. The upregulation of ZO-1 after downregulation of miR-196a or Runx2 is associated with reduced sphere formation and suppressed transwell invasion and transendothelial ability, indicating a critical role for ZO-1 upregulation in suppressing EMT and cancer metastasis.

Overexpression of OPN might lead to intrahepatic metastasis, early recurrence, and poorer prognosis of surgically resected HCC [29,30]. Our study revealed that the level of HoxC8 in the miR-196a-downregulated PLC cells was significantly increased. Thus, miR-196a might not mediate HCC progression through direct modulation of the expression of HoxC8. Runx2, a master transcription factor that controls osteogenesis, can regulate OPN expression through targeting the OPN promoter [31,32]. Our results indicate that miR-196a downregulation results in decreased expression of the Runx family and OPN. Knockdown of Runx2 resulted in similar trends of changes in tumor and stem cell phenotypes compared to miR-196a downregulation. By transient restoration of Runx2 in miR-196a-downregulated HCC in vivo, aggressive tumor phenotypes can be restored. After carefully analyzing the 3′-UTR of Runx2 mRNA, we did not find any seed element (AACUACCUA) of miR-196a. Thus, we reasoned that miR-196a might not directly regulate the post-transcription of Runx2, which is not surprising. Homeobox gene (*Hox*) binding elements (GCTTAATT) are reported to locate in the proximal region of Runx2 promoter [33]. Previous studies revealed the miR-196a was located within the Hox cluster and post-transcriptionally regulated several Hox genes including *HoxC8*, *HoxB8*, and *HoxA2* in the human genome [34]. miR-196a was reported to promote osteoblast differentiation through down-regulating HoxC8 and HoxB8 and further interacting with Smad1 and Smad6 [35]. Runx2 acts as a downstream target of Smad. Although the level of HoxC8 was suggested to correlate with HCC progression [36], miR-196a might not exert its function on Runx2 through modulating HoxC8 expression. As a key regulator of osteogenesis, the expression of Runx2 was carefully regulated, not only by transcription factor, but also by miRNA [37]. There are several miRNAs targeting sequences located in the 3′-UTR of Runx2 [38,39]. Our preliminary data indicated the expression of several miRNAs targeting the 3′-UTR of Runx2 was decreased after knockdown of miR-196a (Figure S5). Our findings suggest that miR-196a mediates HCC progression by reducing the level of miRNAs targeting to the 3′-UTR of Runx2 and subsequently the downstream OPN, followed by activation of EMT regulators and stemness genes which finally lead to increased HCC invasiveness and self-renewal.

Cancer metastasis is a complex process for tumor cells to migrate from the primary site to the distant location and involves multiple steps, including intravasation and extravasation. Cancer cells migrate into vessels, survive in the circulation, migrate again out of vessels, colonize, and proliferate at the secondary site [22]. The transendothelial abilities of HCC were suppressed after miR-196a downregulation. This result implies that there is potential for disruption of critical steps of intravasation and extravasation in cancer metastasis by targeting miR-196a. miR-196a was reported to have strong correlations with tumor stage, poor prognosis, lymph node metastasis, and distant metastasis in colorectal cancer [40]. Herein, our results show that pulmonary metastasis of HCC was significantly suppressed after knockdown of miR-196a. Taken together, miR-196a is a potential target of HCC treatment, especially in those with aggressive cancer behaviors, such as those prone to develop venous invasions. miR-196a can also be exploited for the construction of a synthetic genetic switch to distinguish HCC cells and normal cells for selective killing of HCC cells [41].

## 4. Materials and Methods

### 4.1. Tissue Collection and Total RNA Extraction

Tumor samples with HBV-related HCC were collected by Taiwan Liver Cancer Network. The tissue samples used in our study were delinked and anonymized samples obtained from a biobank (Taiwan Liver Cancer Network). The individual patient inform consent is exempted, and the use of the biobank HCC samples had been approved by the Institutional Review Board of Taipei Veterans General Hospital (IRB No. 97-09-17A and No. 2014-01-003B). All HBV-infected patients were confirmed as positive for serum hepatitis B surface antigen in serum and negative for antibodies of the hepatitis C and delta viruses. The total RNAs were extracted from those tissues according to the manufacturer’s protocols. The extracted RNAs were preserved in liquid nitrogen until use. Purified normal liver tissue RNA (FirstChoice^®^ Human Liver Total RNA, Ambion™ AM7960), certified to contain small RNAs, was purchased from Life Technologies (Carlsbad, CA, USA).

### 4.2. Cell Cultures and Total RNA Extraction of HCC Cell Lines

HCC cell lines including Hep3B (ATCC HB-8064), HepG2 (ATCC HB-8065), Huh7 (JCRB0403), PLC (ATCC CRL-8024), Mahlavu [42], and SNU449 (ATCC CRL-2234) were obtained and used in this study. Cells 1 × 10^4^ were cultured in Dulbecco’s modified Eagle medium (DMEM) culture medium (Life Technologies, Carlsbad, CA, USA) supplemented with 10% fetal bovine serum (FBS, Gibco^®^, Thermo Fisher Scientific Inc., Waltham, MA, USA), 1% nonessential amino acid (Gibco^®^), 1% penicillin–streptomycin (Gibco^®^), and 1% L-glutamine (Gibco^®^) in 100 mm culture dishes. Cells were kept in a humidified incubator at 37 °C with 5% CO_2_ and subcultured every 3–5 days. Cells were harvested 48 h after subculture. Total RNA was extracted using TRIzol reagent (Thermo Fisher Scientific Inc.) according to the manufacturer’s protocol. NanoDrop Spectrophotometers (Thermo Fisher Scientific Inc.) were used to determine the concentration of total RNA, and purified RNA was stored in a −80 °C freezer for future use.

### 4.3. Reverse Transcription and Quantitative Real-Time PCR (RT-qPCR)

The designated probes and primers of miR-196a were optimized and obtained from Exiqon (Vedbaek, Denmark). In the reverse transcription reaction, cDNA was synthesized from purified total RNA using an ExiLERATE LNA cDNA Synthesis kit (ExiLERATE LNA, Exiqon). Quantitative real-time PCR was performed using ExiLERATE LNA qPCR System (Exiqon) according to the manufacturer’s protocol with the LightCycler^®^ 480 System (Roche Applied Science, Mannheim, Germany). Expression data of U6 RNA was assigned as reference. The expression level of miR-196a was normalized and performed using threshold-crossing values obtained by LightCycler 480 software according to manufacturer’s instructions.

### 4.4. Generation of MiR-196a-Knockdown Cell Lines

The miRZip-196a anti-miR-196a miRNA construct (MZIP196a-PA-1, System Biosciences, CA, USA) which produces short, single-stranded anti-miR-196a miRNA was cloned into a lentiviral vector pGreenPuro (System Biosciences). Lentivirus production was performed by transfection of the 293TN Cell Line (System Biosciences) with pPACKH1 packaging systems (System Biosciences). The lentiviral particles were harvested from supernatant 48 h post transfection using ultracentrifugation at 4 °C for 2.5 h. The PLC cells were transduced with anti-miR-196a containing lentiviral particles with a multiplicity of infection (MOI) of 2–4. Stable clones were selected with 4 μg/mL puromycin for 2 weeks. The expression of anti-miR-196a lentiviral vectors on PLC cells was evaluated by green fluorescence under a fluorescence microscope. The efficacy of knockdown of miR-196a was confirmed by RT-qPCR. 

### 4.5. Colony Formation Assay

First, cells were treated with TrypLE trypsin (Gibco^®^, Thermo Fisher Scientific Inc., Waltham, MA, USA) at 37 °C for 10 min to create a single-cell suspension. Cells (3 × 10^3^) were seeded over soft agar for incubation. After 2 weeks, colonies were evaluated under a light microscope, and those with a diameter above 60 µm were counted as positive.

### 4.6. Invasion Assay

The assay was performed using a 24-well BD BioCoat Matrigel Invasion Chamber (BD Biosciences, San Jose, CA, USA), where 1 × 10^4^ cells with the serum-free medium were seeded on the upper chamber, while the bottom chamber was filled with 750 μL of medium with 15% FBS. Then, cells were allowed to migrate for 24 h. Migrated cells in the bottom chamber were stained using Liu’s stain method and counted under a light microscope.

### 4.7. Sphere Formation Assay

Cells were collected with trypsin digestion and centrifuged at 3500 rpm for 5 min at 4 °C, then suspended in a modified tumor sphere medium (DMEM/F12) consisting of a chemically defined serum-free medium with N2 supplement, recombinant human epidermal growth factor (20 ng/mL, PeproTech, Rocky Hill, NJ, USA) and fibroblast growth factor (20 ng/mL, PeproTech). Cells (10^5^) were seeded in a 10 cm Ultra-low dish (Corning, NY, USA) for sphere formation. Cells were incubated with modified tumor sphere medium. Spheres were observed and evaluated under a light microscope. The efficacy was calculated as the percentage of spheres formed per original number of cells seeded.

### 4.8. Transendothelial Invasion Assay

The transendothelial cell assay was performed as described by the manufacturer (CytoSelect Tumor Transendothelial Migration, CBA-216, Cell Biolabs, San Diego, CA, USA). Human umbilical vein endothelial cells (HUVEC, 5 × 10^4^) were cultured for 48 h to form a monolayer of endothelium on top of an extracellular matrix coated porous membrane of the insert chamber. The endothelial monolayer was treated with tumor necrosis factor-alpha. PLC cells (1 × 10^6^ cells/mL) were suspended in serum-free medium and labeled by preincubating the cells with CytoTracker dyes (Cell Biolabs, Inc. San Diego, CA, USA) for 60 min at 37 °C. CytoTracker labeled cells (300 μL) were injected into the prepared insert chambers covered with endothelial cells. The inserts were transferred to bottom chambers containing 500 μL of DMEM with 10% FBS. For the next 24 h, the cells were allowed to migrate through the monolayer of the endothelium. Then, the upper chamber was removed. The tumor cells remaining in the bottom chamber were harvested and were measured by optical density values at 570 nm.

### 4.9. Protein Extraction and Western Blotting

PLC cells were washed twice with ice-cold phosphate-buffered saline (PBS), then the media were removed, and cells were further lysed in lysis buffer in the presence of cocktail protease inhibitors (P-1512, A.G. Scientific, San Diego, CA, USA). The mixture was constantly agitated at 4 °C for 30 min, and the cell debris was removed by centrifuging the samples at 12,000 rpm for 20 min. Protein concentration was determined by the Bradford assay (Bio-Rad, Hercules, CA, USA). Protein samples were denatured at 95 °C for 10 min in Laem mli buffer. Western blotting was performed as follows: the same amounts (20–30 μg) of the denatured protein samples were loaded into each well and separated by 10% sodium dodecyl sulfate–polyacrylamide gel electrophoresis (SDS-PAGE). The separated proteins were further transferred to a polyvinylidene difluoride (PVDF) blotting membrane. Blots were probed with specific primary antibodies, then incubated with appropriate horseradish peroxidase-conjugated secondary antibodies for 1 h. Bands were visualized with enhanced chemiluminescence reagents (NEL122001EA, PerkinElmer, Waltham, MA, USA).

### 4.10. Immunohistochemical Staining

Sections of tissue sample were deparaffinized and rehydrated. Antigen retrieval was performed by heating slides in 10 mM citrate buffer (pH 6.0) for 40 min. Endogenous peroxidase activity was inactivated by 3% hydrogen peroxide. The slides were then blocked with 0.005 g/mL bovine serum albumin (BSA) for 30 min, and primary antibodies were added. After incubating with primary antibodies, the sections were examined using the Super Sensitive IHC detection system (Biogenex, CA, USA) with 3-amino-9-ethylcarbazole (AEC) chromogen and counterstained with hematoxylin. Slides in the absence of primary antibody were used as negative controls.

### 4.11. Immunofluorescence Staining

Immunofluorescence staining was performed as described previously. Briefly, cells (5 × 10^5^/well) were seeded on culture slides (Millicell EZ slide, Millipore, ON, Canada) 1 day before staining. At 80% confluence, cells were washed twice with PBS, fixed with fresh 4% formaldehyde for 15 min, penetrated with 0.1% Triton X-100 in 1× PBS for 5 min, incubated with primary antibody for 1 h, washed, and added with fluorescence-conjugated secondary antibody if necessary. Alex Fluor 488 phalloidin (0.15 µM; cat # A12379), mouse anti-ZO-1 antibody (5 µg/mL; cat # 339100), and SlowFade Gold antifade reagent (cat # S36938) were from Thermo Fisher Scientific Inc. Images were acquired by phase-contrast fluorescence microscopy (DMI3000 B, Leica, Wetzlar, Germany) and processed using Q Capture software program.

### 4.12. In Vivo Tumorigenic and Metastatic Study

NOD/SCID mice (8 weeks old) were maintained under pathogen-free conditions at Fu-Jen Catholic University Animal Center. This study was approved by the Institutional Animal Care and Use Committee (IACUC) of Fu-Jen Catholic University (IACUC No. A10528) and carried out in accordance with the guidelines and regulations of laboratory animals. The tumor growth was evaluated via a subcutaneous xenograft model. An estimated amount of 2 × 10^6^ PLC cells expressing anti-miR-196a miRNA or control vector were subcutaneously implanted into mice. Four mice were used for each cell line, and the growth of the tumor was monitored weekly. Tumors were removed from the animals 7 weeks post-injection or when the mice reached humane endpoints, including morbidity, immobility, unresponsiveness, recumbency, failure to eat or drink, and loss of more than 20% body weight. Tumors were collected, fixed in formalin, measured grossly under a dissecting microscope, and analyzed by hematoxylin and eosin staining.

The in vivo metastasis abilities were examined with a tail vein intravenous injection model. An estimated amount of 2 × 10^5^ PLC cells stably expressing anti-miR-196a miRNA or control vector were injected via tail veins into the mice. Six mice were used for each cell line, and the growth of tumors was monitored for 10 weeks or when the mice reached humane endpoints. The mice lungs were harvested, fixed in 10% formalin, paraffin-embedded, and stained with hematoxylin and eosin according to a standard protocol.

### 4.13. Measurement of Intratumoral Microvessel Density

Angiogenesis was assessed and quantified with the measurement of the formation of three-dimensional tubes within the tumor harvested from in vivo xenograft model. Depending on the size of the sections, 5 to 8 areas within the tumor were randomly selected for evaluation at 100× magnification. These areas were subsequently used to analyze the intratumoral microvessel density measurements at 200× magnification. The microvessel density was measured according to the Weidner method [43]. Each CD31-positive cell cluster of immunoreactivities that contacted the selected field was counted as a single vessel, including the morphologically identifiable vessels with lumens.

### 4.14. Statistical Analysis

The characteristics of the clinical HCC patients were correlated with the expression of miR-196a. The median value of miR-196a expression in all HCC samples was used as a cutoff value to define the higher or lower expression of miR-196a. Independent sample t-test was used to evaluate group differences of continuous variables that passed the normality test, and independent two-group Mann-Whitney U test was performed for continuous variables that failed the normality test. The Chi-square test was utilized to analyze the discrete variables. Univariate and multivariate Cox regression analyses were employed for analyzing the influence of clinical and tumor features, and miR-196a expression in HCC progression and prognosis. Parameters with statistically sound significance (*p* < 0.05) or trend of significance (*p* < 0.2) in univariate analysis were further investigated by multivariate analysis. A Kaplan–Meier method was used for survival analysis comparing the cumulative incidence of overall and disease-free survivals between the study groups. The log-rank test was conducted to compare the difference in overall and disease-free survival distributions between the study groups. All statistical analyses described were performed using the R statistics package [44]. Statistical significance was denied at a two-tailed probability value of less than 0.05 in all analyses.

## 5. Conclusions

In conclusion, this study shows that high expression of miR-196a is significantly associated with progression and postoperative recurrence of HCC in a subset of younger patients. miR-196a mediates HCC progression via upregulation of Runx2, OPN, EMT regulators, and stemness genes. Hence, miR-196a is a potential clinical marker for aggressive HCC and a potential target for HCC treatment.

## Figures and Tables

**Table 1:** Correlation of high and low expression of miR-196a with clinical, pathological, and serological features of patients with hepatocellular carcinoma.

Variable	Total	miR-196a Expression		*p* Value
	(*n* = 83)	High (*n* = 42)	Low (*n* = 41)	
**Patient demographics**				
Age ^1^	51 (44–63.5)	47.5 (43–58.3)	56 (45–64)	0.0369
Male, No (%)	70 (84.3)	35 (83.3)	35 (85.4)	1.0
Cirrhosis, No (%)	38 (45.8)	20 (47.6)	18 (43.9)	0.9049
Diabetes mellitus, No (%)	10 (12.0)	5 (11.9)	5 (12.2)	1.0
**Serum biochemistries** ^1^				
Albumin (g/dL)	4.0 (3.6–4.1)	3.9 (3.4–4.1)	4.0 (3.8–4.3)	0.0386
ALT (IU/L)	43 (34–56.8)	44 (34–65)	40 (34–55)	0.4064
AST (IU/L)	39.5 (30–58.5)	42 (31.5–60.3)	37 (29.8–56.5)	0.4132
Total bilirubin (mg/dL)	0.8 (0.6–1.0)	0.8 (0.6–1.0)	0.8 (0.6–1.0)	0.8798
ALP (IU/L)	84 (62.5–107)	84 (61–103)	83 (65–107)	0.8695
**Viral factors**				
HBeAg (Positive/Negative)	10/46	8/22	2/24	0.1338
HBV genotype (B/C)	49/27	25/14	24/13	1.0
HBV DNA (copies/mL) ^1^	1.96 × 10^5^ (1.41 × 10^4^–1.81 × 10^6^)	2.02 × 10^5^ (1.81 × 10^4^–1.46 × 10^6^)	1.16 × 10^5^ (1.09 × 10^4^–2.06 × 10^6^)	0.9242
**Tumor factors**				
Tumor size (cm) ^1^	4 (2.5–7.3)	4.5 (2.5–8.3)	3.5 (2.5–6.5)	0.2981
AFP (ng/mL), < 20 vs ≥ 20	37/44	12/28	25/16	0.0100
Differentiation (Well/Moderate/Poor)	2/67/14	1/33/8	1/34/6	0.8656
HCC pattern (Solitary/Multiple)	64/19	31/11	33/8	0.6435
Microvascular invasion (Yes/No)	32/51	19/23	13/28	0.298
Macrovascular invasion (Yes/No)	9/74	8/34	1/40	0.0375
Tumor stage (I vs II + III + IV)	42/41	16/26	26/15	0.0369
Follow-up (months) ^1^	42 (26.5–51.5)	40.5 (26.5–47.8)	43 (27–53)	0.3942

^1^ Data are presented with median and interquartile range. AFP: alpha-fetoprotein; ALP: alkaline phosphatase; ALT: alanine aminotransferase; AST: aspartate aminotransferase; HBV: hepatitis B virus; HCC: hepatocellular carcinoma.

**Table 2:** Cox proportional hazard analyses for recurrence of hepatocellular carcinoma.

Variable	Univariate Analyses			Multivariate Analyses		
	Crude HR	95% CI	*p* Value	Adjusted HR	95% CI	*p* Value
**Host factors**						
Age ≥ 50	0.991	0.542–1.811	0.9756			
Male	1.074	0.474–2.434	0.8634			
Cirrhosis	0.990	0.537–1.825	0.973			
Diabetes mellitus	1.238	0.521–2.942	0.629			
**Viral factors**						
Positive HBeAg	0.809	0.306–2.141	0.6694			
HBV DNA ≥ 10^4^ copies/mL	1.978	0.776–5.040	0.1529			
HBV genotype C vs. B	1.585	0.849–2.957	0.1481			
**Tumor factors**						
Tumor size ≥ 5 cm	1.422	0.773–2.613	0.2573			
AFP ≥ 20 ng/mL	1.327	0.711–2.474	0.3741			
Well differentiation	0.893	0.123–6.504	0.9111			
Multinodularity	1.799	0.900–3.595	0.0964			
Microvascular invasion	3.429	1.831–6.419	<0.001	4.582	2.215–9.479	<0.001
Macrovascular invasion	2.196	0.853–5.656	0.1031			
High miR-196a expression	2.124	1.148–3.929	0.016	2.395	1.207–4.752	0.0125

AFP: alpha-fetoprotein; CI: confidence interval; HBV: hepatitis B virus; HR: hazard ratio.

## Supplementary Materials

The following are available online at https://www.mdpi.com/2072-6694/11/10/1549/s1, Figure S1: Relative expression of miR-196a in hepatocellular carcinoma cell lines, Figure S2: miR-196a is required for self-renewal and transwell invasion of SNU449 cells, Figure S3: Higher magnification of the immunohistochemical staining pictures shown in Figure 4B (A), 4D (B) and 5B (C), Figure S4: Runx2 downregulation alters cytoskeleton organization, ZO-1 localization, but not E-cadherin, Figure S5: Knockdown of miR-196a is associated with an increased expression of the miRNAs targeting the 3′-UTR of Runx2 mRNA, Figure S6: Uncropped Western blots corresponding to Figure 5A (A), 5C (B) and 6A (C), Table S1: Correlation of recurrence with clinical, pathological and serological features of patients with hepatocellular carcinoma.
