# Supplementary material for: High Expression of MicroRNA-196a is Associated with Progression of Hepatocellular Carcinoma in Younger Patients
Source: Cancers (Basel). 2019 Oct 13;11(10):1549. doi: 10.3390/cancers11101549 (PMC6826650; doi:10.3390/cancers11101549)
Supplement: Supplementary file 1 [file cancers-11-01549-s001.pdf]

Supplementary Materials: High Expression of MicroRNA-196a is Associated with Progression of Hepatocellular Carcinoma in Younger Patients

Shen-Yung Wang, Chih-Li Chen, Yu-Chen Hu, Yi Chi, Yen-Hua Huang, Chien-Wei Su, Wen-Juei Jeng, Yuh-Jin Liang and Jaw-Ching Wu

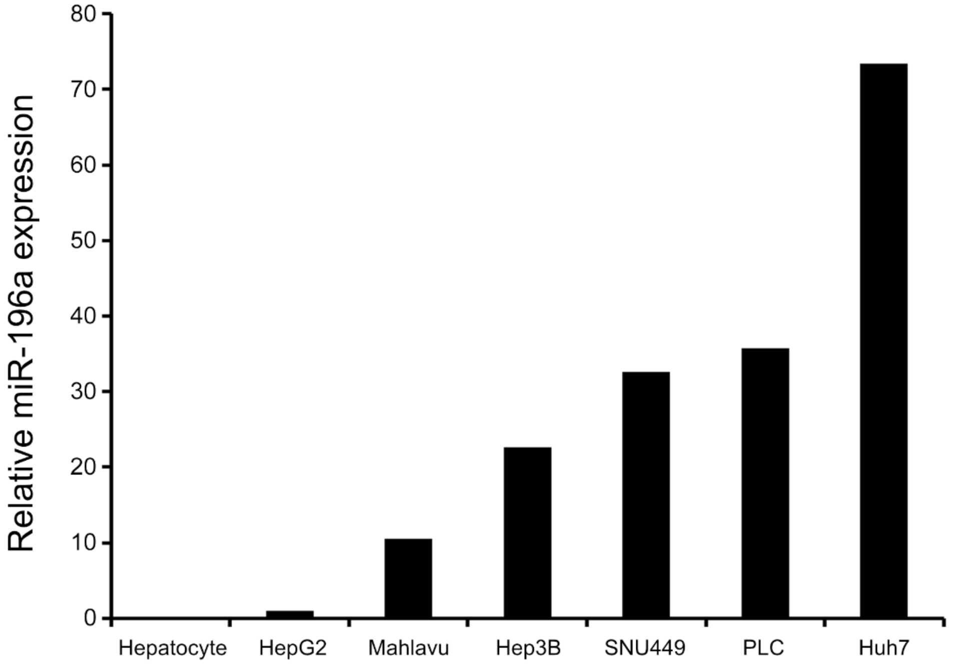

Figure S1. Relative expression of miR-196a in hepatocellular carcinoma cell lines. Relative expression of miR-196a in hepatocellular carcinoma cell lines, including HepG2, Mahlavu, Hep3B, SNU449, PLC, and Huh7. The expression was shown in levels relative to the lowest miR-196a expressed cell line, HepG2.

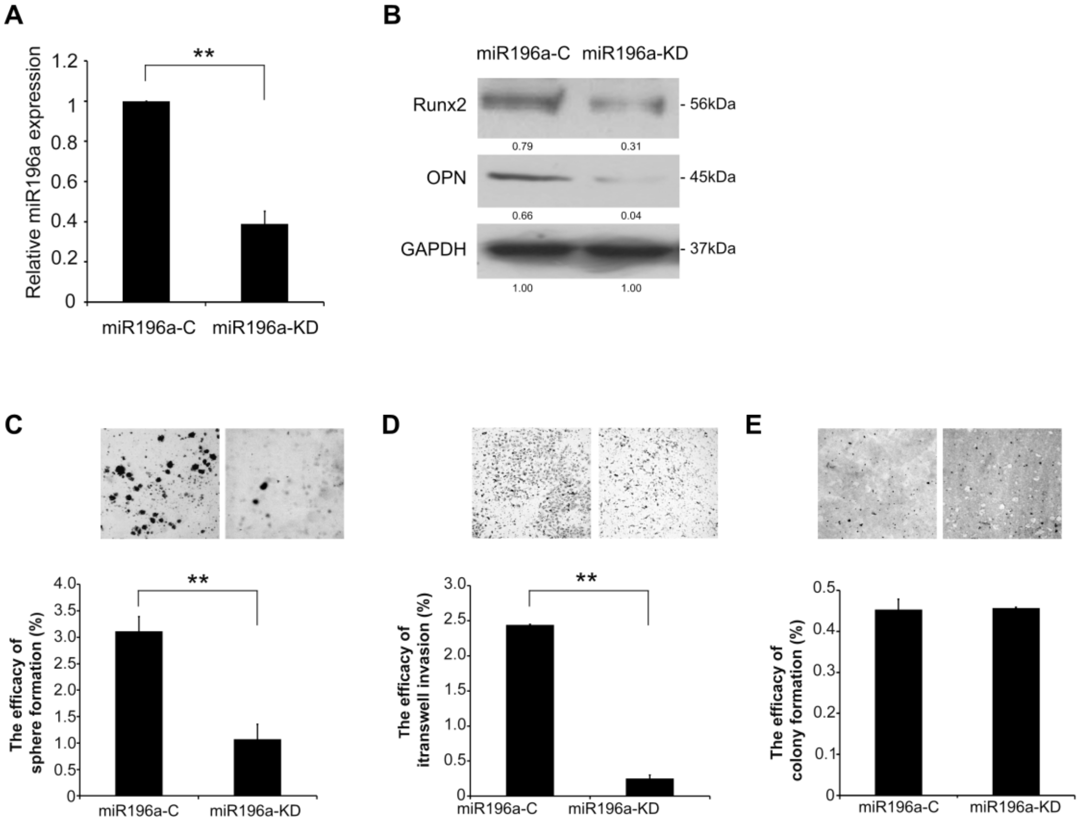

Figure S2. miR-196a is required for self-renewal and transwell invasion of SNU449 cells. (**A**) Downregulation of miR-196a after infection of lentiviral vectors expressing anti-sense miR-196a (miR-196a-KD) compared with that with negative control vectors (miR-196a-C) (**$p < 0.01$). (**B**) Western blot showed that miR-196a downregulation resulted in a decrease of Runx2 and OPN. (**C–E**) Knockdown of miR-196a significantly decreased the sphere formation of (**C**) and invasiveness of (**D**) but not colony formation of SNU449 (**E**) (original magnification, ×50 (**C**), ×100 (**D**), ×25 (**E**), **$p < 0.01$).

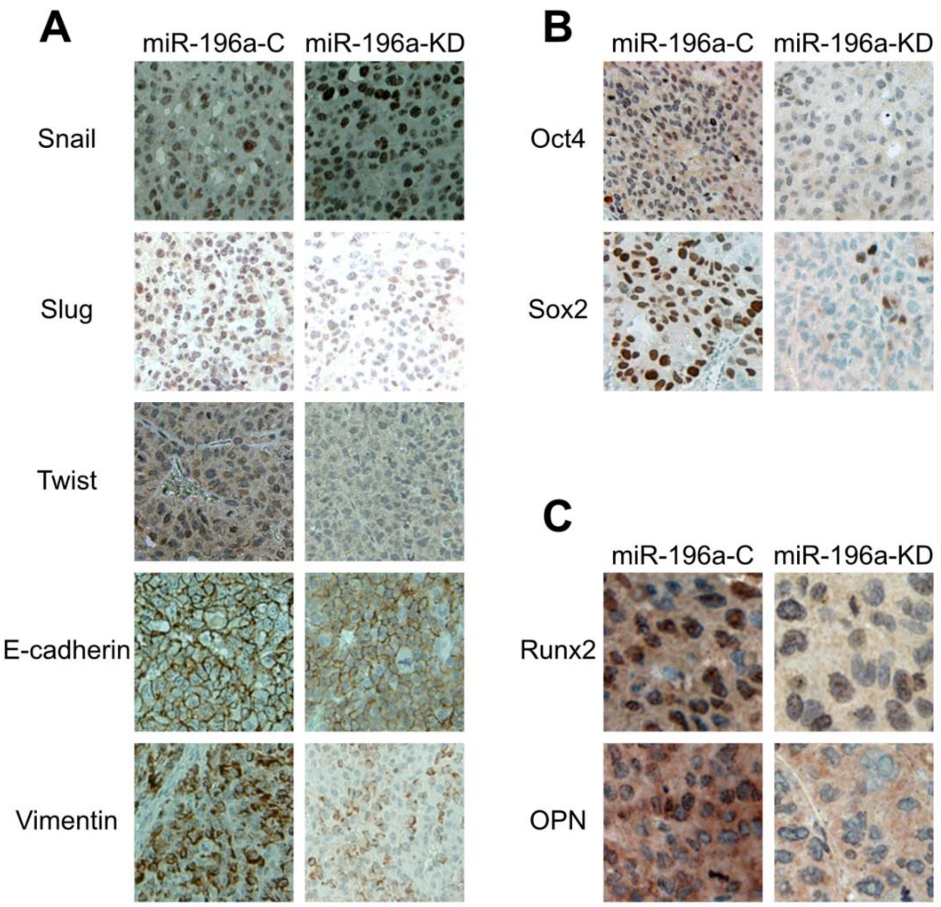

Figure S3. Higher magnification of the immunohistochemical staining pictures shown in Figure 4B (**A**), 4D (**B**) and 5B (**C**). Original magnification, ×200 (**A**), ×200 (**B**), ×400 (**C**).

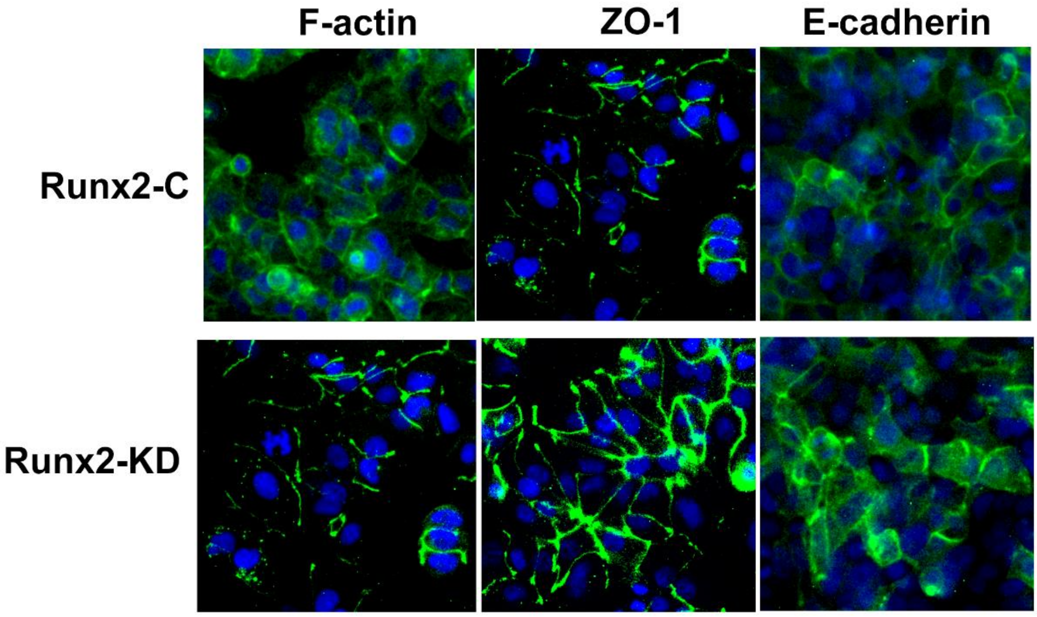

Figure S4. Runx2 downregulation alters cytoskeleton organization, ZO-1 localization, but not E-cadherin. Left: Knockdown of Runx2 (Runx2-KD) reduced stress fiber formation and actin staining in PLC cells. Middle: Runx2 downregulation resulted in higher expression of tight junction-associated protein ZO-1. Right: Expression and localization of E-cadherin were similar in PLC cells with or without Runx2 knockdown. Original magnification, ×400.

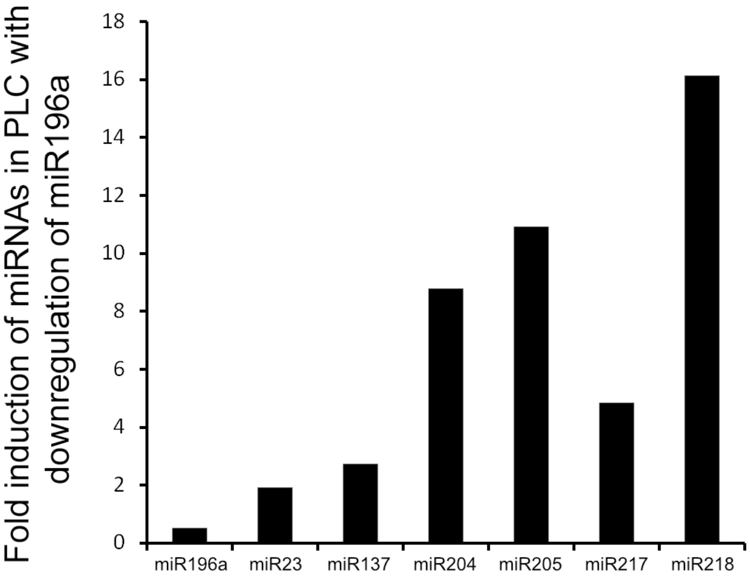

Figure S5. Knockdown of miR-196a is associated with an increased expression of the miRNAs targeting the 3′-UTR of Runx2 mRNA. Several miRNAs including miR-23, miR-137, miR-204, miR-205, miR-217, and miR-218 showed increased expression levels in miR-196a-downregulated PLC compared with those in the negative control.

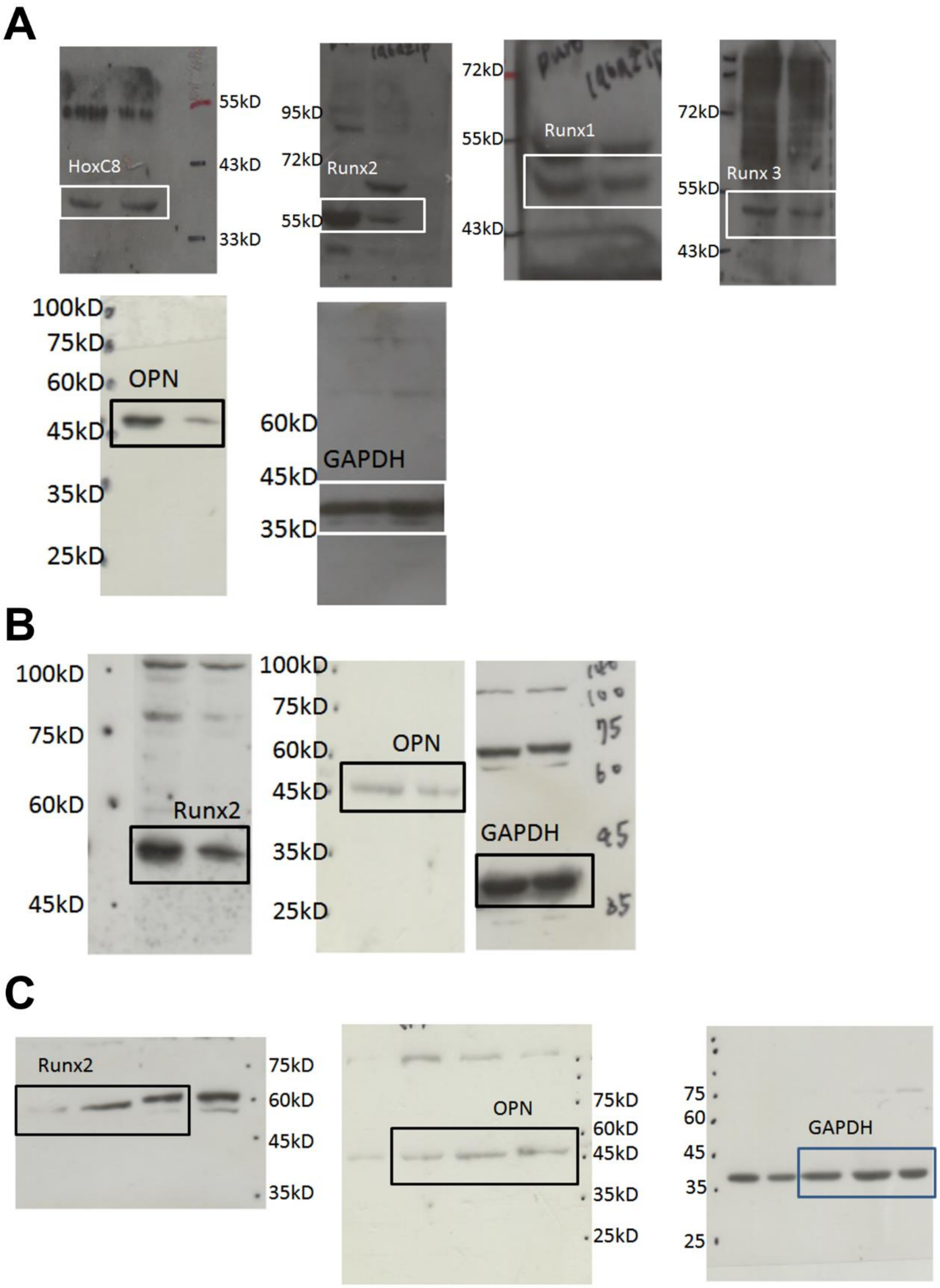

Figure S6. Uncropped Western blots corresponding to Figure 5A (**A**), 5C (**B**) and 6A (**C**).

Table S1. Correlation of recurrence with clinical, pathological and serological features of patients with hepatocellular carcinoma.

Variable	Recurrence		*p* Value
	Yes (*n* = 43)	No (*n* = 34)	
**Patient demographics**			
Age [1]	50 (44–63.5)	52.5 (44–64)	0.955
Male, No (%)	36 (56.3)	28 (43.8)	1.0
Cirrhosis, No (%)	18 (41.9)	14 (41.2)	1.0
Diabetes mellitus, No (%)	6 (14.0)	3 (8.8)	0.7349
**Serum biochemistries** [1]			
Albumin (g/dL)	3.95 (3.6–4.1)	4.0 (3.7–4.2)	0.5555
ALT (IU/L)	45.5 (33–65.8)	38 (34–49)	0.1552
AST (IU/L)	39.5 (32–61)	36.5 (28.5–54)	0.3315
Total bilirubin (mg/dL)	0.8 (0.6–1.0)	0.85 (0.63–1.08)	0.386
ALP (IU/L)	89.5 (57.8–106.2)	78.5 (65.3–107)	0.9196
**Viral factors**			
HBeAg (Positive/Negative)	5/23	4/19	1.0
HBV genotype (B/C)	22/18	24/7	0.087
HBV DNA (copies/ml) [1]	4.99×10^5 (2.53×10^4–3.11×10^6)	9.16×10^4 (1.02×10^4–1.62×10^6)	0.1532
**Tumor factors**			
Tumor size (cm) [1]	4.0 (2.75–8.25)	4.15 (2.5–6.5)	0.5307
AFP (ng/mL), < 20 vs ≥ 20	17/24	16/18	0.8008
Differentiation (Well/Moderate/Poor)	1/26/6	1/25/8	0.5395
HCC pattern (Solitary/Multiple)	32/11	28/6	0.5776
Microvascular invasion (Yes/No)	22/21	8/26	0.0255
Macrovascular invasion (Yes/No)	5/38	2/32	0.6371
High miR-196a expression (Yes/No)	26/17	12/22	0.0495
Tumor stage (I vs II + III + IV)	14/29	24/10	0.0020

[1] Data are presented with median and interquartile range. AFP: alpha-fetoprotein; ALP: alkaline phosphatase; ALT: alanine aminotransferase; AST: aspartate aminotransferase; HBV: hepatitis B virus; HCC: hepatocellular carcinoma.

© 2019 by the authors. Licensee MDPI, Basel, Switzerland. This article is an open access article distributed under the terms and conditions of the Creative Commons Attribution (CC BY) license (http://creativecommons.org/licenses/by/4.0/).
